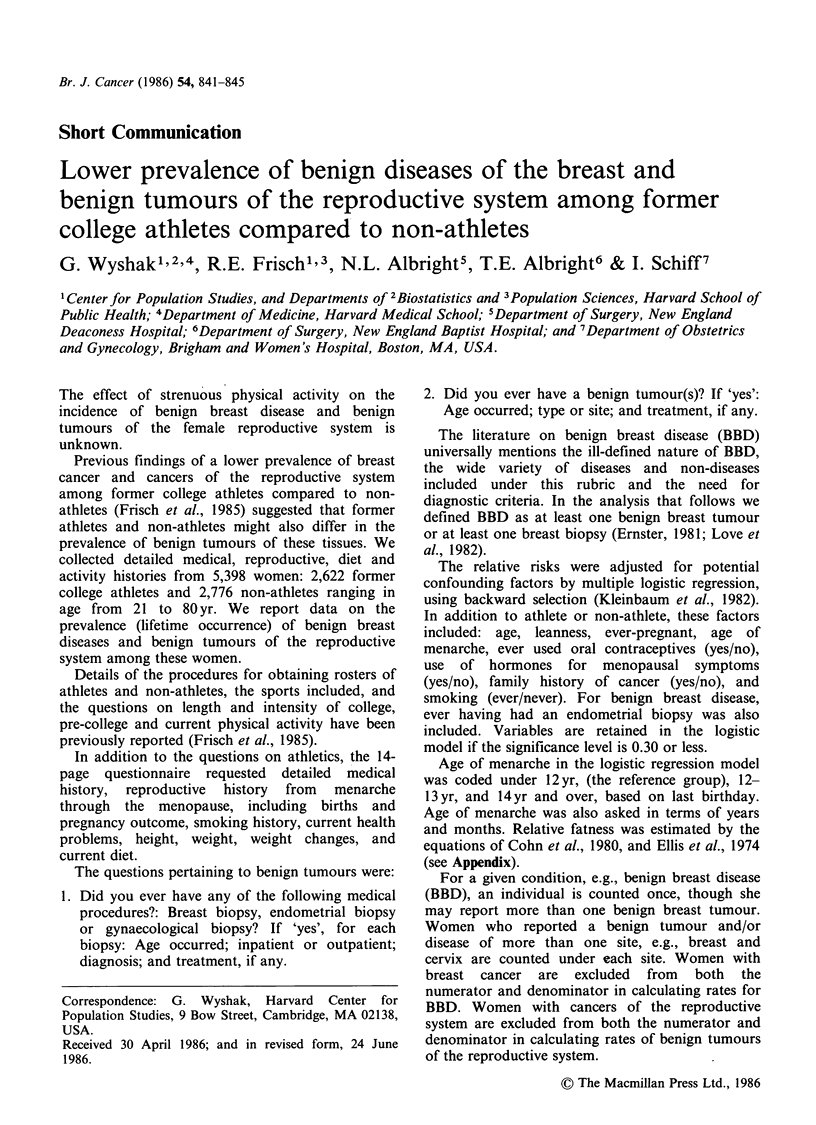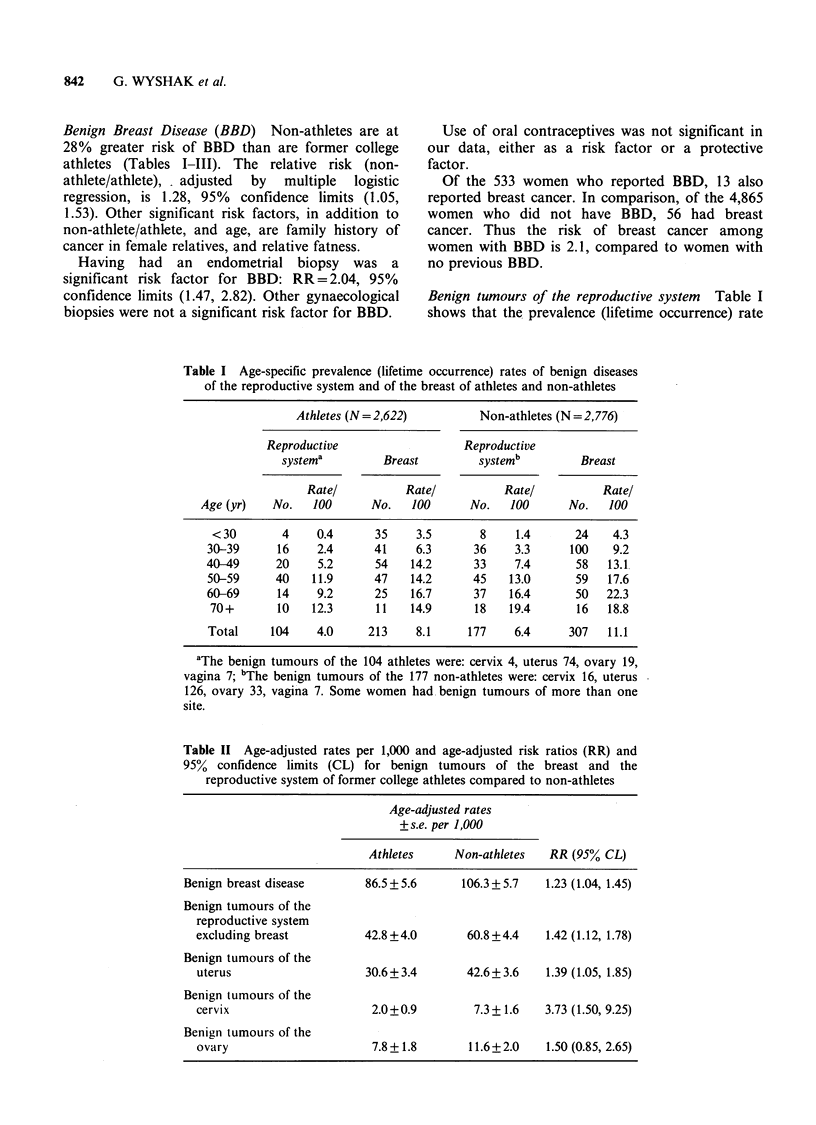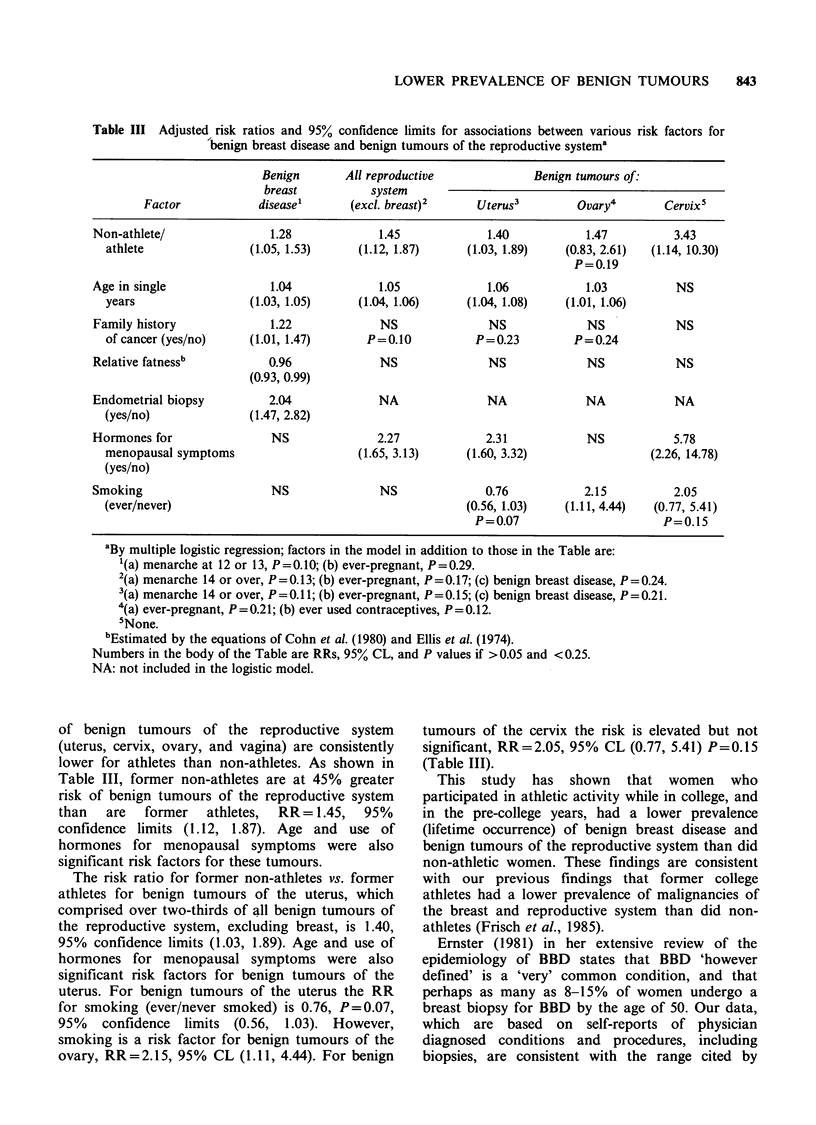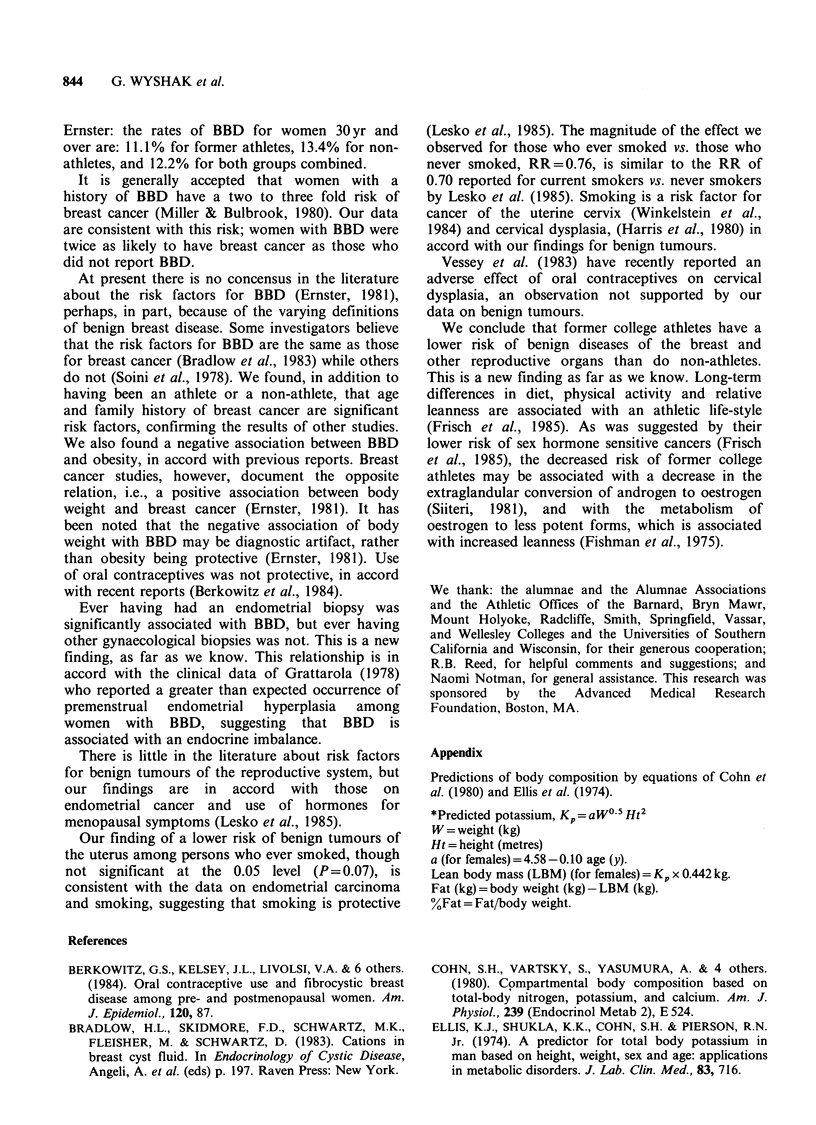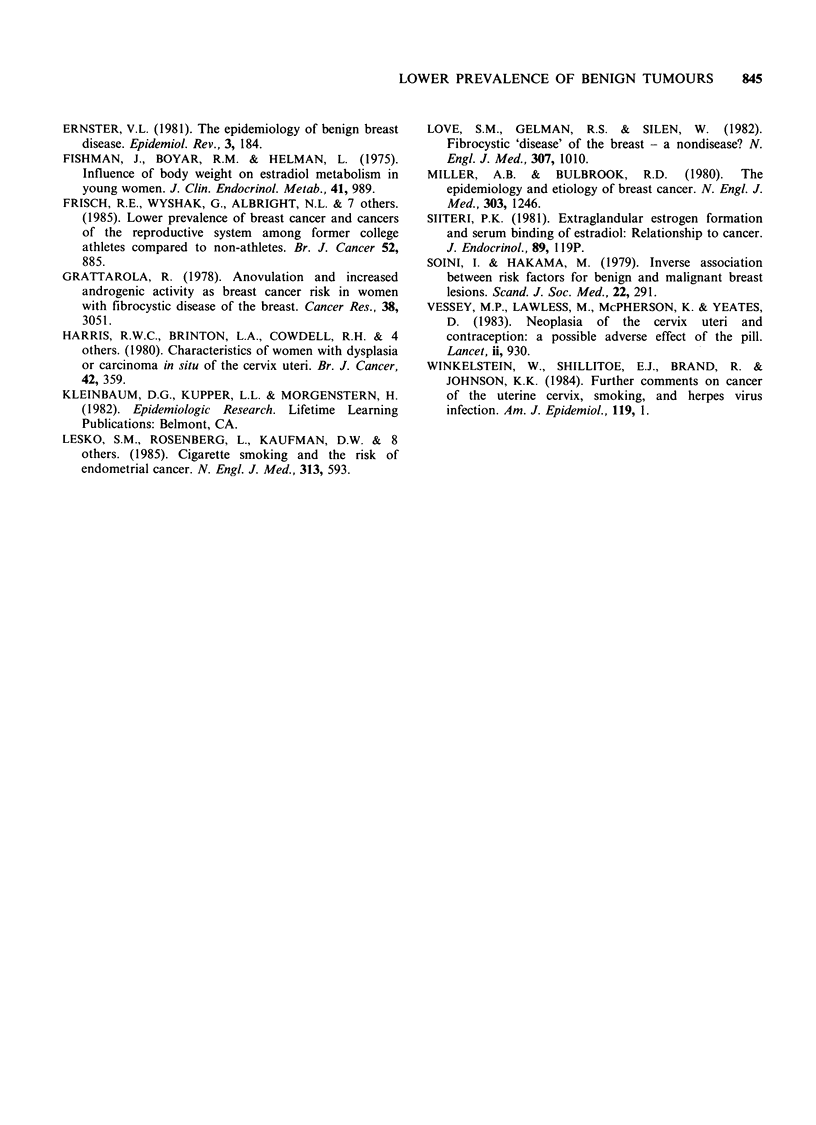# Lower prevalence of benign diseases of the breast and benign tumours of the reproductive system among former college athletes compared to non-athletes.

**DOI:** 10.1038/bjc.1986.249

**Published:** 1986-11

**Authors:** G. Wyshak, R. E. Frisch, N. L. Albright, T. E. Albright, I. Schiff


Br. J. Cancer (1986) 54, 841–845

### Short Communication

### Lower prevalence of benign diseases of the breast and benign tumours of the reproductive system among former college athletes compared to non-athletes

G. Wyshak[1,2,4], R.E. Frisch[1,3], N.L. Albright[5], T.E. Albright[6] & I. Schiff[7]

[1]*Center for Population Studies, and Departments of* [2]*Biostatistics and* [3]*Population Sciences, Harvard School of Public Health;* [4]*Department of Medicine, Harvard Medical School;* [5]*Department of Surgery, New England Deaconess Hospital;* [6]*Department of Surgery, New England Baptist Hospital; and* [7]*Department of Obstetrics and Gynecology, Brigham and Women's Hospital, Boston, MA, USA.*

The effect of strenuous physical activity on the incidence of benign breast disease and benign tumours of the female reproductive system is unknown.

Previous findings of a lower prevalence of breast cancer and cancers of the reproductive system among former college athletes compared to non-athletes (Frisch *et al.*, 1985) suggested that former athletes and non-athletes might also differ in the prevalence of benign tumours of these tissues. We collected detailed medical, reproductive, diet and activity histories from 5,398 women: 2,622 former college athletes and 2,776 non-athletes ranging in age from 21 to 80 yr. We report data on the prevalence (lifetime occurrence) of benign breast diseases and benign tumours of the reproductive system among these women.

Details of the procedures for obtaining rosters of athletes and non-athletes, the sports included, and the questions on length and intensity of college, pre-college and current physical activity have been previously reported (Frisch *et al.*, 1985).

In addition to the questions on athletics, the 14-page questionnaire requested detailed medical history, reproductive history from menarche through the menopause, including births and pregnancy outcome, smoking history, current health problems, height, weight, weight changes, and current diet.

The questions pertaining to benign tumours were:

1. Did you ever have any of the following medical procedures?: Breast biopsy, endometrial biopsy or gynaecological biopsy? If 'yes', for each biopsy: Age occurred; inpatient or outpatient; diagnosis; and treatment, if any.
2. Did you ever have a benign tumour(s)? If 'yes': Age occurred; type or site; and treatment, if any.

The literature on benign breast disease (BBD) universally mentions the ill-defined nature of BBD, the wide variety of diseases and non-diseases included under this rubric and the need for diagnostic criteria. In the analysis that follows we defined BBD as at least one benign breast tumour or at least one breast biopsy (Ernster, 1981; Love *et al.*, 1982).

The relative risks were adjusted for potential confounding factors by multiple logistic regression, using backward selection (Kleinbaum *et al.*, 1982). In addition to athlete or non-athlete, these factors included: age, leanness, ever-pregnant, age of menarche, ever used oral contraceptives (yes/no), use of hormones for menopausal symptoms (yes/no), family history of cancer (yes/no), and smoking (ever/never). For benign breast disease, ever having had an endometrial biopsy was also included. Variables are retained in the logistic model if the significance level is 0.30 or less.

Age of menarche in the logistic regression model was coded under 12 yr, (the reference group), 12–13 yr, and 14 yr and over, based on last birthday. Age of menarche was also asked in terms of years and months. Relative fatness was estimated by the equations of Cohn *et al.*, 1980, and Ellis *et al.*, 1974 (see **Appendix**).

For a given condition, e.g., benign breast disease (BBD), an individual is counted once, though she may report more than one benign breast tumour. Women who reported a benign tumour and/or disease of more than one site, e.g., breast and cervix are counted under each site. Women with breast cancer are excluded from both the numerator and denominator in calculating rates for BBD. Women with cancers of the reproductive system are excluded from both the numerator and denominator in calculating rates of benign tumours of the reproductive system.

Correspondence: G. Wyshak, Harvard Center for Population Studies, 9 Bow Street, Cambridge, MA 02138, USA.
Received 30 April 1986; and in revised form, 24 June 1986.

© The Macmillan Press Ltd., 1986

*Benign Breast Disease* (*BBD*) Non-athletes are at 28% greater risk of BBD than are former college athletes (Tables I–III). The relative risk (non-athlete/athlete), adjusted by multiple logistic regression, is 1.28, 95% confidence limits (1.05, 1.53). Other significant risk factors, in addition to non-athlete/athlete, and age, are family history of cancer in female relatives, and relative fatness.

Having had an endometrial biopsy was a significant risk factor for BBD: RR=2.04, 95% confidence limits (1.47, 2.82). Other gynaecological biopsies were not a significant risk factor for BBD.

Use of oral contraceptives was not significant in our data, either as a risk factor or a protective factor.

Of the 533 women who reported BBD, 13 also reported breast cancer. In comparison, of the 4,865 women who did not have BBD, 56 had breast cancer. Thus the risk of breast cancer among women with BBD is 2.1, compared to women with no previous BBD.

*Benign tumours of the reproductive system* Table I shows that the prevalence (lifetime occurrence) rate

**Table I** Age-specific prevalence (lifetime occurrence) rates of benign diseases of the reproductive system and of the breast of athletes and non-athletes

| | *Athletes* (*N*=*2,622*) | | | | Non-athletes (N=*2,776*) | | | |
|---|---|---|---|---|---|---|---|---|
| | *Reproductive system*[a] | | *Breast* | | *Reproductive system*[b] | | *Breast* | |
| *Age* (*yr*) | *No.* | *Rate/100* | *No.* | *Rate/100* | *No.* | *Rate/100* | *No.* | *Rate/100* |
| <30 | 4 | 0.4 | 35 | 3.5 | 8 | 1.4 | 24 | 4.3 |
| 30–39 | 16 | 2.4 | 41 | 6.3 | 36 | 3.3 | 100 | 9.2 |
| 40–49 | 20 | 5.2 | 54 | 14.2 | 33 | 7.4 | 58 | 13.1 |
| 50–59 | 40 | 11.9 | 47 | 14.2 | 45 | 13.0 | 59 | 17.6 |
| 60–69 | 14 | 9.2 | 25 | 16.7 | 37 | 16.4 | 50 | 22.3 |
| 70+ | 10 | 12.3 | 11 | 14.9 | 18 | 19.4 | 16 | 18.8 |
| Total | 104 | 4.0 | 213 | 8.1 | 177 | 6.4 | 307 | 11.1 |

[a]The benign tumours of the 104 athletes were: cervix 4, uterus 74, ovary 19, vagina 7; [b]The benign tumours of the 177 non-athletes were: cervix 16, uterus 126, ovary 33, vagina 7. Some women had benign tumours of more than one site.

**Table II** Age-adjusted rates per 1,000 and age-adjusted risk ratios (RR) and 95% confidence limits (CL) for benign tumours of the breast and the reproductive system of former college athletes compared to non-athletes

| | *Age-adjusted rates ±s.e. per 1,000* | | |
|---|---|---|---|
| | *Athletes* | *Non-athletes* | *RR* (*95% CL*) |
| Benign breast disease | 86.5±5.6 | 106.3±5.7 | 1.23 (1.04, 1.45) |
| Benign tumours of the reproductive system excluding breast | 42.8±4.0 | 60.8±4.4 | 1.42 (1.12, 1.78) |
| Benign tumours of the uterus | 30.6±3.4 | 42.6±3.6 | 1.39 (1.05, 1.85) |
| Benign tumours of the cervix | 2.0±0.9 | 7.3±1.6 | 3.73 (1.50, 9.25) |
| Benign tumours of the ovary | 7.8±1.8 | 11.6±2.0 | 1.50 (0.85, 2.65) |

**Table III** Adjusted risk ratios and 95% confidence limits for associations between various risk factors for benign breast disease and benign tumours of the reproductive system[a]

| Factor | Benign breast disease[1] | All reproductive system (excl. breast)[2] | Benign tumours of: Uterus[3] | Ovary[4] | Cervix[5] |
|---|---|---|---|---|---|
| Non-athlete/ athlete | 1.28 (1.05, 1.53) | 1.45 (1.12, 1.87) | 1.40 (1.03, 1.89) | 1.47 (0.83, 2.61) $P=0.19$ | 3.43 (1.14, 10.30) |
| Age in single years | 1.04 (1.03, 1.05) | 1.05 (1.04, 1.06) | 1.06 (1.04, 1.08) | 1.03 (1.01, 1.06) | NS |
| Family history of cancer (yes/no) | 1.22 (1.01, 1.47) | NS $P=0.10$ | NS $P=0.23$ | NS $P=0.24$ | NS |
| Relative fatness[b] | 0.96 (0.93, 0.99) | NS | NS | NS | NS |
| Endometrial biopsy (yes/no) | 2.04 (1.47, 2.82) | NA | NA | NA | NA |
| Hormones for menopausal symptoms (yes/no) | NS | 2.27 (1.65, 3.13) | 2.31 (1.60, 3.32) | NS | 5.78 (2.26, 14.78) |
| Smoking (ever/never) | NS | NS | 0.76 (0.56, 1.03) $P=0.07$ | 2.15 (1.11, 4.44) | 2.05 (0.77, 5.41) $P=0.15$ |

[a]By multiple logistic regression; factors in the model in addition to those in the Table are:
[1](a) menarche at 12 or 13, $P=0.10$; (b) ever-pregnant, $P=0.29$.
[2](a) menarche 14 or over, $P=0.13$; (b) ever-pregnant, $P=0.17$; (c) benign breast disease, $P=0.24$.
[3](a) menarche 14 or over, $P=0.11$; (b) ever-pregnant, $P=0.15$; (c) benign breast disease, $P=0.21$.
[4](a) ever-pregnant, $P=0.21$; (b) ever used contraceptives, $P=0.12$.
[5]None.
[b]Estimated by the equations of Cohn *et al.* (1980) and Ellis *et al.* (1974).
Numbers in the body of the Table are RRs, 95% CL, and $P$ values if $>0.05$ and $<0.25$.
NA: not included in the logistic model.

of benign tumours of the reproductive system (uterus, cervix, ovary, and vagina) are consistently lower for athletes than non-athletes. As shown in Table III, former non-athletes are at 45% greater risk of benign tumours of the reproductive system than are former athletes, RR = 1.45, 95% confidence limits (1.12, 1.87). Age and use of hormones for menopausal symptoms were also significant risk factors for these tumours.

The risk ratio for former non-athletes *vs.* former athletes for benign tumours of the uterus, which comprised over two-thirds of all benign tumours of the reproductive system, excluding breast, is 1.40, 95% confidence limits (1.03, 1.89). Age and use of hormones for menopausal symptoms were also significant risk factors for benign tumours of the uterus. For benign tumours of the uterus the RR for smoking (ever/never smoked) is 0.76, $P=0.07$, 95% confidence limits (0.56, 1.03). However, smoking is a risk factor for benign tumours of the ovary, RR = 2.15, 95% CL (1.11, 4.44). For benign tumours of the cervix the risk is elevated but not significant, RR = 2.05, 95% CL (0.77, 5.41) $P=0.15$ (Table III).

This study has shown that women who participated in athletic activity while in college, and in the pre-college years, had a lower prevalence (lifetime occurrence) of benign breast disease and benign tumours of the reproductive system than did non-athletic women. These findings are consistent with our previous findings that former college athletes had a lower prevalence of malignancies of the breast and reproductive system than did non-athletes (Frisch *et al.*, 1985).

Ernster (1981) in her extensive review of the epidemiology of BBD states that BBD 'however defined' is a 'very' common condition, and that perhaps as many as 8–15% of women undergo a breast biopsy for BBD by the age of 50. Our data, which are based on self-reports of physician diagnosed conditions and procedures, including biopsies, are consistent with the range cited by

Ernster: the rates of BBD for women 30 yr and over are: 11.1% for former athletes, 13.4% for non-athletes, and 12.2% for both groups combined.

It is generally accepted that women with a history of BBD have a two to three fold risk of breast cancer (Miller & Bulbrook, 1980). Our data are consistent with this risk; women with BBD were twice as likely to have breast cancer as those who did not report BBD.

At present there is no concensus in the literature about the risk factors for BBD (Ernster, 1981), perhaps, in part, because of the varying definitions of benign breast disease. Some investigators believe that the risk factors for BBD are the same as those for breast cancer (Bradlow *et al.*, 1983) while others do not (Soini *et al.*, 1978). We found, in addition to having been an athlete or a non-athlete, that age and family history of breast cancer are significant risk factors, confirming the results of other studies. We also found a negative association between BBD and obesity, in accord with previous reports. Breast cancer studies, however, document the opposite relation, i.e., a positive association between body weight and breast cancer (Ernster, 1981). It has been noted that the negative association of body weight with BBD may be diagnostic artifact, rather than obesity being protective (Ernster, 1981). Use of oral contraceptives was not protective, in accord with recent reports (Berkowitz *et al.*, 1984).

Ever having had an endometrial biopsy was significantly associated with BBD, but ever having other gynaecological biopsies was not. This is a new finding, as far as we know. This relationship is in accord with the clinical data of Grattarola (1978) who reported a greater than expected occurrence of premenstrual endometrial hyperplasia among women with BBD, suggesting that BBD is associated with an endocrine imbalance.

There is little in the literature about risk factors for benign tumours of the reproductive system, but our findings are in accord with those on endometrial cancer and use of hormones for menopausal symptoms (Lesko *et al.*, 1985).

Our finding of a lower risk of benign tumours of the uterus among persons who ever smoked, though not significant at the 0.05 level ($P=0.07$), is consistent with the data on endometrial carcinoma and smoking, suggesting that smoking is protective (Lesko *et al.*, 1985). The magnitude of the effect we observed for those who ever smoked *vs.* those who never smoked, RR = 0.76, is similar to the RR of 0.70 reported for current smokers *vs.* never smokers by Lesko *et al.* (1985). Smoking is a risk factor for cancer of the uterine cervix (Winkelstein *et al.*, 1984) and cervical dysplasia, (Harris *et al.*, 1980) in accord with our findings for benign tumours.

Vessey *et al.* (1983) have recently reported an adverse effect of oral contraceptives on cervical dysplasia, an observation not supported by our data on benign tumours.

We conclude that former college athletes have a lower risk of benign diseases of the breast and other reproductive organs than do non-athletes. This is a new finding as far as we know. Long-term differences in diet, physical activity and relative leanness are associated with an athletic life-style (Frisch *et al.*, 1985). As was suggested by their lower risk of sex hormone sensitive cancers (Frisch *et al.*, 1985), the decreased risk of former college athletes may be associated with a decrease in the extraglandular conversion of androgen to oestrogen (Siiteri, 1981), and with the metabolism of oestrogen to less potent forms, which is associated with increased leanness (Fishman *et al.*, 1975).

We thank: the alumnae and the Alumnae Associations and the Athletic Offices of the Barnard, Bryn Mawr, Mount Holyoke, Radcliffe, Smith, Springfield, Vassar, and Wellesley Colleges and the Universities of Southern California and Wisconsin, for their generous cooperation; R.B. Reed, for helpful comments and suggestions; and Naomi Notman, for general assistance. This research was sponsored by the Advanced Medical Research Foundation, Boston, MA.

**Appendix**

Predictions of body composition by equations of Cohn *et al.* (1980) and Ellis *et al.* (1974).

*Predicted potassium, $K_p = aW^{0.5}Ht^2$
$W$ = weight (kg)
$Ht$ = height (metres)
$a$ (for females) = 4.58 − 0.10 age ($y$).
Lean body mass (LBM) (for females) = $K_p \times 0.442$ kg.
Fat (kg) = body weight (kg) − LBM (kg).
%Fat = Fat/body weight.

**References**

BERKOWITZ, G.S., KELSEY, J.L., LIVOLSI, V.A. & 6 others. (1984). Oral contraceptive use and fibrocystic breast disease among pre- and postmenopausal women. *Am. J. Epidemiol.*, **120,** 87.

BRADLOW, H.L., SKIDMORE, F.D., SCHWARTZ, M.K., FLEISHER, M. & SCHWARTZ, D. (1983). Cations in breast cyst fluid. In *Endocrinology of Cystic Disease*, Angeli, A. *et al.* (eds) p. 197. Raven Press: New York.

COHN, S.H., VARTSKY, S., YASUMURA, A. & 4 others. (1980). Compartmental body composition based on total-body nitrogen, potassium, and calcium. *Am. J. Physiol.*, **239** (Endocrinol Metab 2), E 524.

ELLIS, K.J., SHUKLA, K.K., COHN, S.H. & PIERSON, R.N. Jr. (1974). A predictor for total body potassium in man based on height, weight, sex and age: applications in metabolic disorders. *J. Lab. Clin. Med.*, **83,** 716.

ERNSTER, V.L. (1981). The epidemiology of benign breast disease. *Epidemiol. Rev.*, **3**, 184.

FISHMAN, J., BOYAR, R.M. & HELMAN, L. (1975). Influence of body weight on estradiol metabolism in young women. *J. Clin. Endocrinol. Metab.*, **41**, 989.

FRISCH, R.E., WYSHAK, G., ALBRIGHT, N.L. & 7 others. (1985). Lower prevalence of breast cancer and cancers of the reproductive system among former college athletes compared to non-athletes. *Br. J. Cancer* **52**, 885.

GRATTAROLA, R. (1978). Anovulation and increased androgenic activity as breast cancer risk in women with fibrocystic disease of the breast. *Cancer Res.*, **38**, 3051.

HARRIS, R.W.C., BRINTON, L.A., COWDELL, R.H. & 4 others. (1980). Characteristics of women with dysplasia or carcinoma *in situ* of the cervix uteri. *Br. J. Cancer*, **42**, 359.

KLEINBAUM, D.G., KUPPER, L.L. & MORGENSTERN, H. (1982). *Epidemiologic Research*. Lifetime Learning Publications: Belmont, CA.

LESKO, S.M., ROSENBERG, L., KAUFMAN, D.W. & 8 others. (1985). Cigarette smoking and the risk of endometrial cancer. *N. Engl. J. Med.*, **313**, 593.

LOVE, S.M., GELMAN, R.S. & SILEN, W. (1982). Fibrocystic 'disease' of the breast – a nondisease? *N. Engl. J. Med.*, **307**, 1010.

MILLER, A.B. & BULBROOK, R.D. (1980). The epidemiology and etiology of breast cancer. *N. Engl. J. Med.*, **303**, 1246.

SIITERI, P.K. (1981). Extraglandular estrogen formation and serum binding of estradiol: Relationship to cancer. *J. Endocrinol.*, **89**, 119P.

SOINI, I. & HAKAMA, M. (1979). Inverse association between risk factors for benign and malignant breast lesions. *Scand. J. Soc. Med.*, **22**, 291.

VESSEY, M.P., LAWLESS, M., McPHERSON, K. & YEATES, D. (1983). Neoplasia of the cervix uteri and contraception: a possible adverse effect of the pill. *Lancet*, **ii**, 930.

WINKELSTEIN, W., SHILLITOE, E.J., BRAND, R. & JOHNSON, K.K. (1984). Further comments on cancer of the uterine cervix, smoking, and herpes virus infection. *Am. J. Epidemiol.*, **119**, 1.